# On to Nashville

**Published:** 1897-07

**Authors:** 


### SOCIETY PROCEEDINGS.

### ON TO NASHVILLE.

THOSE members of the Association or profession located in the eastern central States radiating at New York, Philadelphia, Baltimore, or Washington will find the way to Nashville prepared for them by the Baltimore & Ohio Railroad, and the following rates may be availed of and the journey made a pleasant outing and the jaunt added to by the following pleasing plans: A ten days' excursion ticket may be purchased in New York for $10, in Philadelphia for $6 to Washington, the national capital. Those from New York may leave that city on Wednesday, September 2d; those from Philadelphia on Tuesday morning, September 3d, at 8.15 o'clock, and remain in the city of Washington until Thursday night at 11.20 o'clock. A special twenty-day excursion ticket is sold from Washington to Nashville and return for $21.05, making the cost from New York $31.05, from Philadelphia $27.05. Leaving Washington at night, this train will reach Chattanooga at 7.40 P.M. the following day, where a stop-over until 11.35 P.M. is made, giving an opportunity for driving over the city for some four hours, arriving at Nashville the morning of the second day, September 6, 1897, at 6.40 o'clock. Monday may be spent at the exposition; Tuesday, Wednesday and Thursday at the Convention; Friday and Saturday at the exposition, leaving Nashville on Saturday evening and arriving home on Monday the 14th.

This trip is by way of Shenandoah, and affords a beautiful ride through that wonderfully interesting and picturesque country. A special season excursion-ticket may be purchased at Washington for $34.75, good until the close of the exposition. Sleepers are attached to these trains both going and coming.

A paper will be presented at Nashville by Dr. E. B. Ackerman, of Brooklyn, N. Y.; also one by Dr. Wm. Dougherty, of Baltimore, Md., whose practical contributions to the Association have earned for their author a very warm appreciation by the members.

"Our Milk-supply," by Dr. Chas. Ellis, of St. Louis, Mo., and a statistical paper upon the "Effects of Tuberculin Injections into Healthy Cattle," by Dr. T. D. Hinebauch, of North Dakota, associated with an important contribution by Dr. Leonard Pearson, of Pennsylvania, will well cover one of the foremost topics of the day.

Knocking at the doors of the Association for admission at Nashville will be found some of the strongest members of the profession over our land, and this meeting will prove a rich return for the grand work done at Buffalo.

Secretary Stewart never tires in his work, and we fear his lamp burns all night for the Association's interest. Secretaries are born, not developed, and our present officer is one of those naturally well-fitted for such a place. He will leave a record to be proud of.

Headquarters will be maintained at the Hotel Tulane. An assembly-room seating two hundred has been offered for our use by the hotel proprietors, with committee-rooms, etc., for the convenience of the delegates. This hotel is maintained on the European plan, rooms $1 per day and upward; regular meals 50 cents each. This hotel has already entertained most of the associations that have convened at Nashville. Hotel accommodations may be secured in advance by writing Dr. T. W. Scott, 537 Broad Street, Nashville, Tenn.

St. Louis will send a large delegation of her veterinarians to Nashville. From all over the South and Southwest signs of increasing interest in our coming meeting continue to enlarge. It is very much hoped for that more of the western ladies will visit Nashville, that they may meet many of those who are planning to attend from the eastern and central States. This proved one of the very brightest points of our Buffalo meeting, and added much to the pleasure of all who were present. There are some new ones further up North that should be seen at Nashville—even as far north as Minnesota.

Sections in "Sanitary Science," "Veterinary Education," and "Practice" are strongly urged for one day at our Nashville meeting. The Executive Committee and officers should take this matter up.

